# Trampolining of Droplets on Hydrophobic Surfaces Using Electrowetting

**DOI:** 10.3390/mi13030345

**Published:** 2022-02-22

**Authors:** Zhantao Wang, Xiaojuan Liu, Li Wang, Cunlu Zhao, Danfeng Zhou, Jiazheng Wei

**Affiliations:** 1 School of Mechanical & Electronic Engineering, Zhongshan Polytechnic, Zhongshan 528400, China; albert212@nwsuaf.edu.cn; 2 School of Information Engineering, Zhongshan Polytechnic, Zhongshan 528400, China; wangli_oet@163.com; 3 Key Laboratory of Thermo-Fluid Science and Engineering of MOE, Xi’an Jiaotong University, Xi’an 710049, China; mclzhao@mail.xjtu.edu.cn; 4 Maglev Engineering Center, National University of Defense Technology, Changsha 410073, China; zhoudanfeng@nudt.edu.cn

**Keywords:** droplet, trampolining, electrowetting, capacitor sensor

## Abstract

Droplet detachment from solid surfaces is an essential part of many industrial processes. Electrowetting is a versatile tool for handling droplets in digital microfluidics, not only on plain surface but also in 3-D manner. Here, we report for the first time droplet trampolining using electrowetting. With the information collected by the real-time capacitor sensing system, we are able to synchronize the actuation signal with the spreading of the droplet upon impacting. Since electrowetting is applied each time the droplet impacts the substrate and switched off during recoiling of the droplet, the droplet gains additional momentum upon each impact and is able to jump higher during successive detachment. We have modelled the droplet trampolining behavior with a periodically driven harmonic oscillator, and the experiments showed sound agreement with theoretical predictions. The findings from this study will offer valuable insights to applications that demands vertical transportation of the droplets between chips arranged in parallel, or detachment of droplets from solid surfaces.

## 1. Introduction

Detaching liquid droplets or liquid separation from solid surface plays an important role in various industrial processes such as self-cleaning [1], heat transfer [1], fuel cells [2] and droplet levitation from cold solid surfaces [3]. A (super)hydrophobic surface is frequently used for the afore-mentioned applications due to the notably reduced solid–liquid adhesion and high contact angles. Several approaches, including droplets coalescence [4], vibration [5], substrate heating [6,7], etc., can be used for droplet detachment from hydrophobic surface, among which electrowetting offers the advantage of higher efficiency and easy controllability [8,9]. Electrowetting (EW) is the means of modifying the wettability of a liquid droplet on a surface by applying an electric field between the surface and the liquid droplet [10]. The electric double-layer capacitor forming at the droplet-substrate interface reduces the effective solid-liquid interfacial tension and changes the apparent contact angle. Direct EW on electrode is limited by the narrow range of contact angle modulation and frequently occurring electrolysis of the droplet. Such limitations are overcome by introducing a dielectric layer between the electrode and the conductive droplet, which is named electrowetting on dielectric (EWOD) [11]. The dielectric layer increases the range of applicable voltage and magnitude of contact angle modulation. Due to its high efficiency and versatility, EWOD has attracted increasing interest in lab-on-a-chip [12], liquid lens [13] and displays technology [14]. The application of electrowetting in lab-on-a-chip systems is named digital microfluidics (DMF), where electrowetting is used for manipulating discrete droplets on patterned electrodes, such as droplet disposal, transportation, merging, splitting and mixing [15]. Recently there have been more and more reports about using electrowetting as a means for droplet detachment and 3-D digital microfluidics [8,16,17,18,19,20,21,22,23,24,25,26]. In electrowetting, a droplet normally gains additional surface area through deformation while a voltage is applied. Such deformation means additional surface energy when the voltage is removed and the apparent contact angle recovered. When the gain of surface energy by the droplet is sufficient enough to overcome adhesion and friction, the droplet will detach from the solid surface [27]. Various groups have studied the EW-induced droplet jumping via experimental and theoretical means, with some focusing on establishing the criterion for droplet detachment [17,18,28] and others more on the application perspective [16,20]. In addition, existing results suggest that comparing with DC voltage, AC voltage is more efficient in detaching the droplet because the dynamic oscillations accompanying AC actuation create more interfacial and kinetic energy [29]. Very recently, by studying the detachment of droplet from a substrate with finite wettability using both experiment and simulations, Cavalli et al. [30] identified the critical droplet volume for EW-induced droplet detachment. Ashraful et al. [31] numerically studied the voltage level, pulse width and droplet volume in affecting the droplet detachment and revealed the synchronous relationship between base diameter and jumping height of the droplet. By studying the droplet jumping on superhydrophobic surface in both air and oil ambient, Wang et al. showed that the droplet jumping height does not increase with the spreading extent monotonically, and a proper pulse time is crucial in achieving a higher jumping [8]. Therefore, modulation of the driving signal becomes very important in efficiently detaching a droplet [32].

In applications that require repetitive vertical transport of the droplet between two plates, monitoring the droplet position becomes very important because this information is useful for timing the electric actuation for each droplet. This is most likely carried out by applying electrowetting only in the spreading phase during the droplet’s impacting process with one plate, to avoid conflict between inertia and electrowetting force. Among the various means for detecting droplet position, the capacitive sensor appears to be the most efficient and convenient one. In DMF, capacitance measurement-based droplet sensing was first demonstrated by Gong et al. [33] and Chen et al. [34], for detecting droplet at desired locations, and followed by Shih et al. [35] for feedback control of the droplets. Murran et al. [36] and Biddut et al. [37] further demonstrate that droplet position between two adjacent electrodes (including co-planar electrodes) can be effectively detected by using capacitance sensor. Since the droplet readily forms a capacitor with the electrodes underneath and the dielectric layer in between, integrating a capacitive sensor into a DMF device becomes quite convenient.

Although droplet jumping by electrowetting has been extensively studied by various researchers, so far, according to our knowledge, there have been very few reports on trampolining a droplet using electrowetting. Actually achieving droplet trampolining via electrowetting offers obvious advantage over the previously reported droplet trampolining that relies on creating low pressure environment^3^ or heating the substrate [38,39,40] to a very high temperature (Leidenfrost drops) and therefore demanding an excessive energy input. In this report, we showed for the first time that EW can be used for trampolining a droplet on superhydrophobic surface. The capacitive sensor is integrated in the DMF chip and used for monitoring the droplet-substrate impact, and therefore, electric actuation can be synchronized with the spreading phase of the impacting process (where the droplet capacitor increases with the droplet-substrate overlapping area), in order to achieve higher jumping and trampolining of the droplet. By combining the gravitational energy with electrical energy, the droplet is shown to rebound higher (restitution coefficient larger than 1) each time it impacts the substrate. With the help of previous studies [3,41], we showed that droplet trampolining can be soundly described using a driven damped droplet-spring model.

## 2. Experiments and Methods

**Figure 1:**
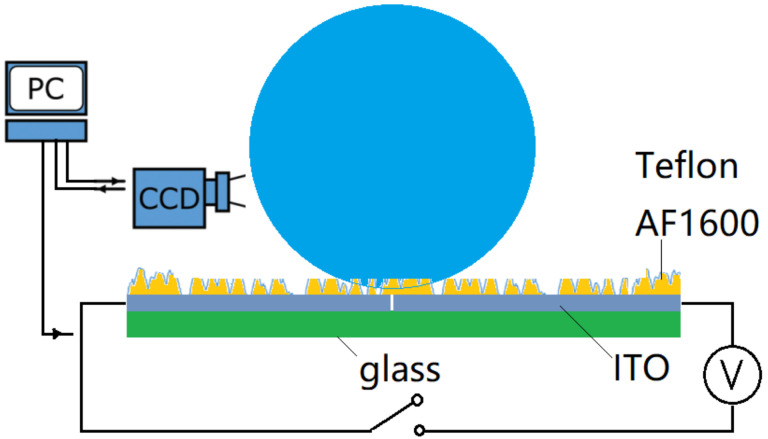
Experimental setup for electrowetting-assisted droplet jumping and trampolining. Note that the Teflon layer has been plasma etched to form superhydrophobic nano structures on top.

The experimental setup (Figure 1) includes the superhydrophobic substrates with coplanar electrodes, the electrowetting system and the video recording system. The superhydrophobic substrates consist of a 1 cm × 2 cm glass slide coated with indium tin oxide (ITO) and the dielectric polytetrafluoroethylene (PTFE/teflon) layer. The sputtered ITO layer on glass was lithographically etched through the middle, creating two electrodes divided by a gap of ~25 µm width. A thin layer (3 µm) of Teflon was dip-coated on the ITO as the dielectric. The dielectric layer was then plasma etched to create superhydrophobic nano structures, following the technique proposed by Ryu et al. [42]. A microcontroller Atmel SAMD21G18B-A precisely outputs low analog voltage (0~5 V) through a low voltage serial to high voltage parallel converter (HV507) with microcontroller SPI interface. The video recording system consists of a Photron SA5 high speed camera, the PC and the backlight source. The camera was used to capture the motion of the droplet from side view at a frame rate of 2000 frames/s. An optic fiber was used as a back light for the side view.

For systems with the co-planar electrodes, if the gap width dividing the electrodes is much smaller comparing with the size of electrodes, the Young–Lippman equation was then modified to Equation (1) for prediction of the contact angle modulation under a given voltage [43].

(1) $$\eta =cos\theta_{U}-cos\theta_{0}=\varepsilon_{eff}\varepsilon_{0}U^{2}/8\gamma d$$

where $\varepsilon_{eff}\varepsilon_{0}$ represents the effective dielectric constant of the Teflon layer, and d its thickness; γ is the surface tension of water and *U* the applied voltage; $\theta_{U}$ and $\theta_{0}$ represent the contact angle of water droplet forms on the substrate with and without voltage applied, and η is the electrowetting number. The behavior of droplet impacting a hydrophobic substrate has been modelled as a damped harmonic oscillator [44,45,46], and the impact time or contact time $t_{c}$ is essentially in the same order with the inertia-capillary time, as

(2) $$t_{c}\sim \sqrt{\frac{m}{\gamma }}={\left(\frac{4\rho R_{0}^{3}}{3\gamma }\right)}^{1/2}$$

where ρ and $R_{0}$ are density and equivalent radius of the droplet. These results were adopted in this paper for the similarity of the system being studied.

**Figure 2:**
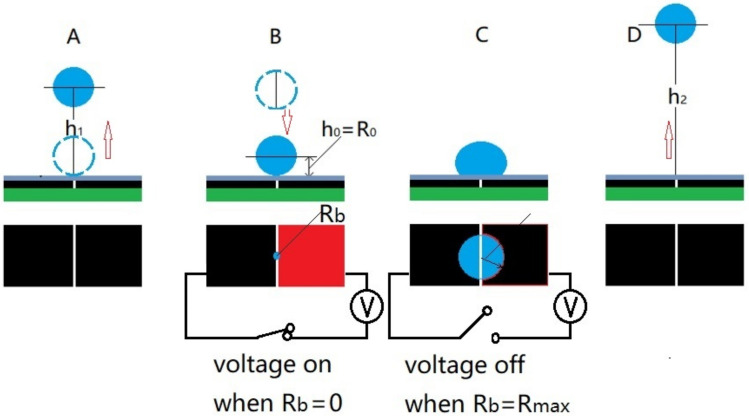
Schematic diagram of trampolining the droplet using electrowetting. With (**A**–**D**) showing the 1st jumping, impacting, spreading, and 2nd detaching of the droplet. The red and dark colors in (**B**) represent the driving and reference electrodes.

Figure 2 shows the schematic diagram for the EW-actuated droplet trampolining. The droplet initially rests on the substrate with its centroid projection falling on the gap between the electrodes (applying a voltage of a few volts can help position the droplet on the gap due to energy minimization principle discussed later). A voltage pulse is first applied to the electrodes and induces the droplet to jump to a height h_1_ (A), when it falls and touches the substrate (electrodes), the pulse (voltage) is applied to the electrodes again (B) to enhance the spreading of the droplet. The pulse continues until the spherical-cap-shaped droplet spreads to its maximum base radius (Rb), and then, the pulse is switched off. This is because under a fixed amplitude of the voltage, a longer pulse does not necessarily lead to a larger spreading of the droplet due to oscillation of the droplet (for this, please see the droplet spreading under different pulse duration in File S3 of the Supplementary Material). The droplet then recoils under surface tension and jumps to a larger height h_2_ (D). The actuation cycle is repeated, and the droplet shows a trampolining behavior on the superhydrophobic surface.

**Figure 3:**
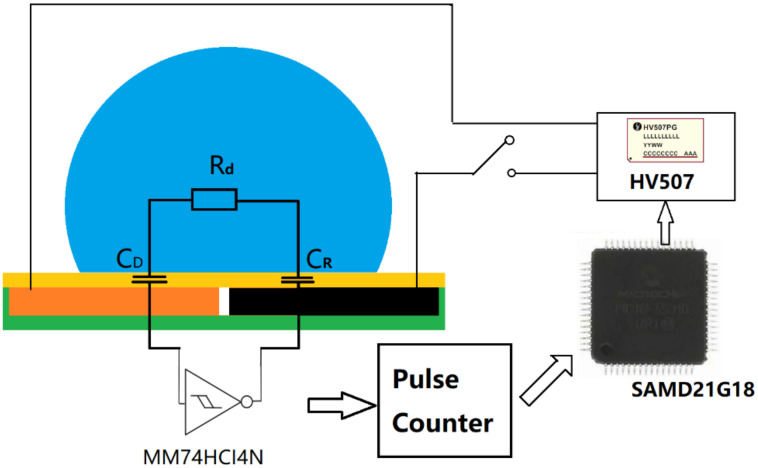
Schematic diagram showing the capacitance measurement circuitry. C_D_ and C_R_ are the capacitor generated by the droplet with the driving and reference electrodes.

To realize the mechanism described in Figure 2, it is very important for us to monitor the moment of the impact and recoiling events of the droplet. For this purpose, we used a droplet-dielectric-electrode-based capacitor. Capacitor sensors have been previously used for detecting the droplet position and speed on a DMF device [37]. In this study, we integrated a droplet capacitive position sensor as illustrated in Figure 3. The equivalent capacitance formed by the co-planar electrodes, the droplet and the dielectric layer in between can be calculated as [43]

(3) $$C_{eq}=\frac{\varepsilon_{0}\varepsilon_{S}}{d}\left(\frac{A_{d}}{A_{t}}\frac{A_{r}{}^{2}}{A_{d}+A_{r}}+\frac{A_{r}}{A_{t}}\frac{A_{d}{}^{2}}{A_{d}+A_{r}}\right)$$

where $\varepsilon_{0}$, $\varepsilon_{S}$ and d are the permittivity of vacuum, the relative permittivity and thickness of the dielectric layer. $A_{d}$, $A_{r}$ and $A_{t}$ are the overlapping areas of the driving and reference electrodes with the droplet.

According to the energy minimization principle, in experiments employing the co-planar electrodes, the droplet will adjust its position while the voltage is on, so that its base covers a same area of the driving and reference electrodes (for this, please refer to Video 5 in File S6 of the Supplementary Material). While the gap is very thin and its area can be neglected, this means $A_{d}=A_{r}=\frac{1}{2}A_{b}$, while $A_{b}$ is the base area of the droplet forming with the substrate excluding the gap area. Indeed, this was observed in our previous experiments of EW-induced droplet jumping. During the subsequent falling and bouncing process, the droplet has repeatedly fell on the same spot providing that the substrate is carefully placed in the horizontal position. This simplifies the problem, since under this circumstance, the capacitator scales linearly with the base area. Equation (3) can be simplified into

(4) $$C_{eq}=\frac{1}{4}\frac{\varepsilon_{0}\varepsilon_{S}}{t}A_{b}=\frac{\pi }{4}\frac{\varepsilon_{0}\varepsilon_{S}}{t}R_{b}^{2}$$

where $R_{b}$ is the base radius of the cap-shaped droplet.

Under such circumstance, the equivalent capacitor (*C_eq_*) formed between the droplet and the electrodes is supposed to scale with the overlapping (footprint) area of the two, and therefore, the capacitor can be conveniently used as a sensor for monitoring the droplet motion, especially for recording the scenario of droplet’s impacting the substrate and the subsequent spreading and recoiling. The moment the capacitor starts to increase indicates touching of the substrate by the droplet (B), and the moment the capacitor reaches its maximum and starts to decrease marks the decrease of the substrate-droplet overlapping area, which also indicates the droplet has started the recoiling process (C). EW are applied to enhance the spreading of the droplet, so in order to avoid the competition between electrowetting-enhanced spreading and inertia driven recoiling, actuation should only be applied when $C_{eq}^{\prime }>0$, namely, the spreading phase of the impacting process. Again, we need to emphasize that under a certain voltage amplitude increasing the AC pulse duration does not necessarily lead to further spreading of the droplet. The capacitor sensor-based feedback system was built for such purpose.

As shown in Figure 3, the capacitor sensor-based feedback system works in the following manner. A microcontroller Atmel SAMD21G18B-A outputs low analog voltage (0–5 V) through a low voltage serial to high voltage parallel converter (HV507) with microcontroller SPI interface. The pulse counter port in the microcontroller is connected with a ring oscillator which measures capacitance on the electrodes, which is also controlled by the microcontroller. By using a fast 20 MHz microcontroller, one cycle of sensing and driving can be completed within 1 ms [33].

The recorded video of droplet motion was analyzed using a self-written program in MATLAB [8]. First, the droplet contour was extracted from the recorded images using MATLAB. By treating the droplet as an axisymmetric revolving body, its shape was decomposed into a number of shape modes (n = 10 in our case) and expressed as a linear combination of the Legendre polynomials as

(5) $$R\left(t,\;\theta \right)=R_{0}+\overset{\infty }{\underset{n=0}{\sum }}c_{n}\left(t\right)P_{n}\left(cos\theta \right)$$

where $R_{0}=({\left(3V/\left(4\pi \right)\right)}^{1/3}$ is the equivalent radius of the droplet with a volume of *V* = 1 uL. $P_{n}\left(cos\theta \right)$ is the Legendre polynomial of mode n, and $c_{n}\left(t\right)$ is the coefficient of mode *n*. The MATLAB program also extracts the position, velocity and acceleration of the centre of mass.

## 3. Results and Discussion

### 3.1. EW-Induced Droplet Jumping

For wettability characterization, a 1 μL (Ro~0.62 mm) Milli-Q water droplet is deposited on the superhydrophobic substrate, which created a contact angle of ~162° with negligible hysteresis (<4 degrees). Then the plasma-etched substrate surface was characterized using tapping mode Atomic Force Microscopy. The obtained image revealed sharp nanostructures (nano-hills) as shown in File S1 of the Supplementary Material. These nano-hills in combination with the essential hydrophobicity of PTFE endowed the substrate surface with superhydrophobicity.

A droplet can be detached from a superhydrophobic surface by using AC or DC pulsatile actuations. Similar to the detachment of a droplet upon coalescence [47,48], it is the translation of surface energy into kinetic energy that drives the droplet to detach from the substrate. Since the droplet starts from a resting state with a contact angle close to 180°, we simplify its surface energy into that of a sphere, which is geometrically the shape with the lowest interface area under volume conservation, and the deviation in interface energy as a result of this approximation is ~1% (please refer to File S2 of the Supplementary Material for the calculation of spherical cap interface energy). By assuming that the droplet recovers its contact angle ($\theta_{0}\sim \pi$) completely when switching off the voltage and neglects dissipation, the excess energy for translation into kinetic energy and eventually gravity energy is

(6) $$E_{exc}=E_{surf-deformed}-E_{sphere}=\gamma \left(S_{deformed}-S_{sphere}\right)\;$$

**Figure 4:**
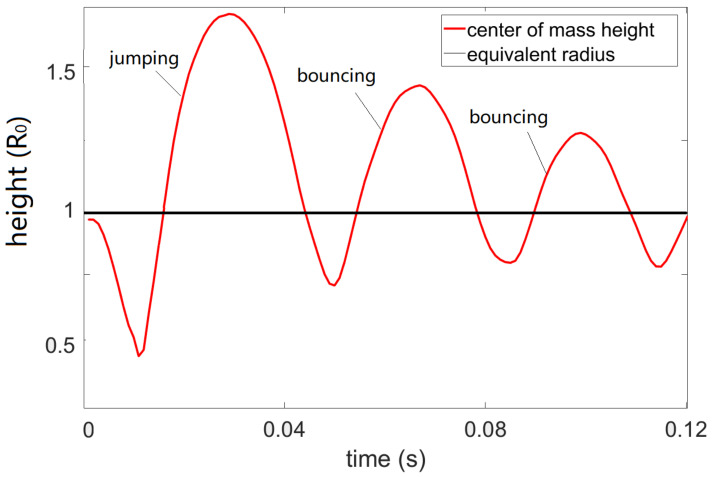
Droplet ($R_{0}=0.62\;\mathrm{mm}$) centroid position as a function of time during one EW-induced jumping cycle; the applied AC signal is a 4 ms AC pulse (sine wave) of 300 V and 10 KHz; the grey line marks the equivalent radius of the droplet from volume conservation. Note that only the first detachment comes from EW actuation, and the subsequent ones indicate bouncing of the droplet on the substrate with a restitution coefficient smaller than 1.

If this additional surface energy overcomes adhesion and friction, the droplet is likely to detach from the solid substrate, and the expected jumping height of the droplet should be approximately

(7) $$\Delta H=\left(E_{exc}-E_{dis}\right)/mg\;$$

where $E_{dis}$ denotes the energy lost via various forms such as internal viscous damping and contact line friction. Figure 4 shows the centroid position as a function of time during the EW-induced jumping motion where an AC voltage of 300 V is applied between the electrodes and a spherical droplet with equivalent radius $R_{0}$ of ~0.85 mm (movie 1 in Supplementary Material). After turning off the voltage, the droplet jumps to a height of ~1.7 $R_{0}$. The droplet continues to bounce off the substrate after impacting the surface, showing a restitution coefficient of ~0.89.

For the droplet jumping experiment, pulses of different duration were applied. The variation of the droplet jumping height with the pulse lengths was recorded and analyzed (for droplet jumping height under different voltage and pulse durations please refer to Figure S4 in the Supplementary Material). We have shown that the evolution of the droplet jumping height under AC pulses of different durations has exhibited periodic damping, resembling the behavior of a damped harmonic oscillator. Moreover, by combining timed actuation with the inertia of the droplet, the translation efficiency from interfacial potential energy to kinetic energy can be optimized [8]. However, due to energy dissipation via internal viscous damping and contact line friction, only 20~30% of the gained interfacial energy can be translated into gravitational energy [8]. Details regarding the energy transfer process during EW-induced droplet jumping and tips for optimizing the pulse duration can be found in our previous publication [8]. Naturally a droplet bounces off the substrate with a restitution coefficient ε<1; however, in this study, we have developed strategies that help us achieve trampolining (ε>1) of the droplet using electrowetting, and this is achieved with the feedback system built in the capacitor sensor.

### 3.2. Droplet Monitoring Using Capacitor Sensor

**Figure 5:**
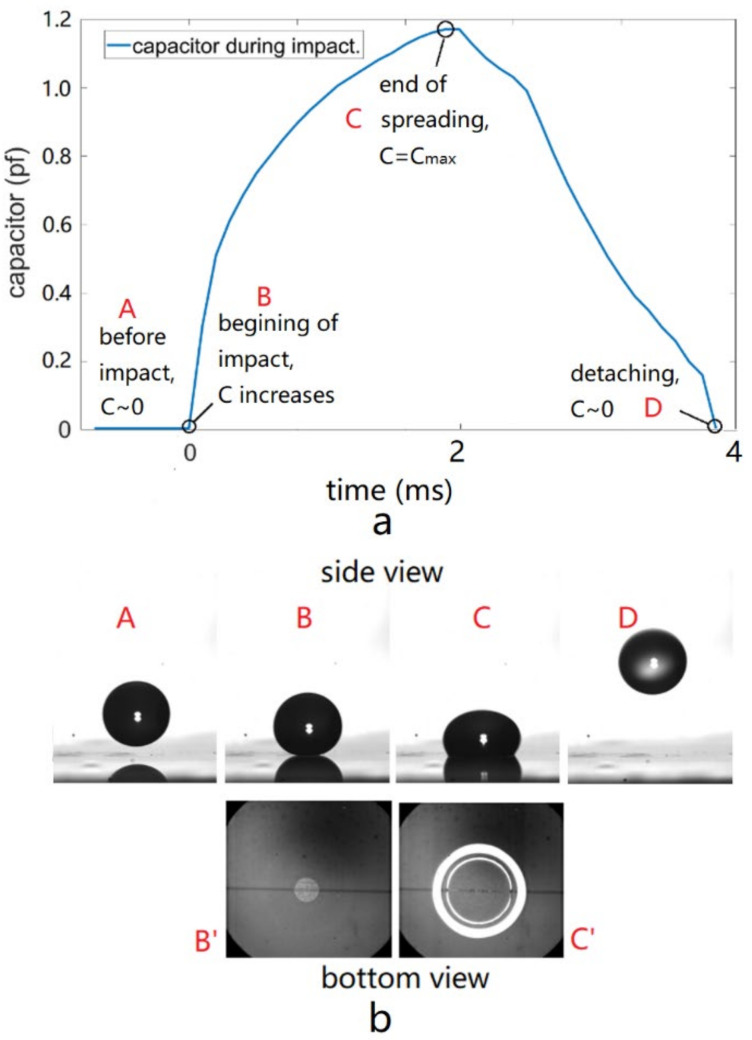
Evolution of the droplet capacitance value during the impacting process (**a**) and the corresponding droplet images at the distinct turning point (**b**). A, B, C and D indicate that the droplet levitates in the air, impacts the substrate, spreads to maximum base radius and leaves the substrate. Note Figure 5b also shows the bottom-view images corresponding to B and C.

Real-time droplet position monitoring plays a key role in EW-induced trampolining of the droplet, and the capacitor sensor gives us the direct information regarding the droplet-substrate impacting process. Figure 5a shows the evolution of the capacitance for the droplet-dielectric-electrodes capacitor during one impacting cycle, where A-B, B-C and C-D mark the falling, impacting and spreading, and recoiling and detaching phases. The images in Figure 5b show the snapshots from side view and bottom view of the fast speed camera; the bottom views of the droplet flying phase (A and D) were not shown because they offer little information. For a droplet falling from height H_1_, the capacitor it forms together with the electrode it overlaps and the dielectric layer in between starts increasing from its minimum initial value ($C_{0}\sim 0$ after normalization) at the instant of impacting the substrate, and that is the point where the electric pulse should be switched on. The actuation pulse lasts until the droplet reaches its maximum base radius and starts to recoil (again due to droplet oscillation under AC actuation this recoiling can happen while the voltage is still on), the pulse is then switched off. For a droplet resting on the substrate, the only difference is that the droplet starts from a point where its capacitance has a non-zero value corresponding with its initial base area, and Figure 5 only depicts the former situation.

Once the droplet jumps to a height H_1_ and falls to the superhydrophobic substrate, it is natural that it will bounce off but to a height smaller than H_1_ due to energy dissipation, which means it has a restitution coefficient smaller than 1 (Figure 4). However, by injecting additional momentum into the droplet once it falls and hits the substrate, it is expected to bounce off to a height larger than H_1_. Indeed, by timing the actuation with the help of the feedback system built on the capacitor sensor, we have achieved trampolining of the droplet.

**Figure 6:**
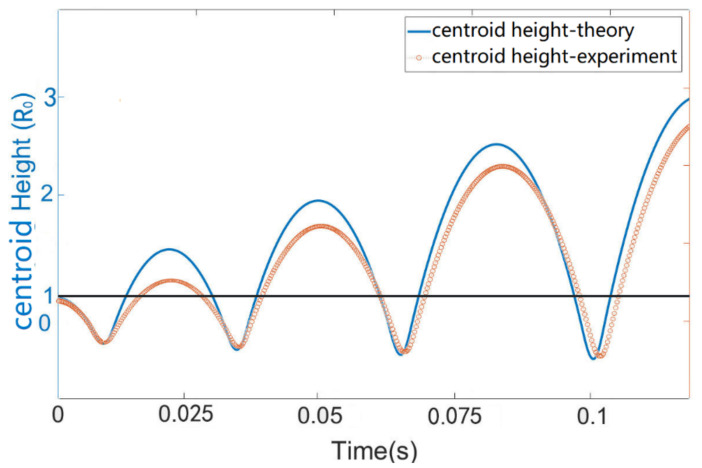
Centroid height ($H_{CM},\;Y\;\mathrm{axis}$) as a function of time for the droplet trampolining on a superhydrophobic surface, driven by electrowetting force applied during the spreading phase of each impact cycle; the applied AC signal is a 200 V sine wave of 10 KHz. Red dotted line represents the experimental results, and the blue solid line represents the theoretical values according to the driven droplet spring model as discussed in Section 3.3. The dark line represents the equilibrium radius ($\;R_{0}\sim 0.62\;\mathrm{mm}$) of the 1 uL droplet. The dots above and below the dark line indicate the flying and impacting phases, respectively, and the time from impacting ($H_{CM}=0$) to recoiling ($H_{CM}^{\prime }=0$) marks the duration of the pulse.

It is seen from the Supplementary Video (movie 2 of File S6 in the Supplementary Materials) that the trampolining of the droplet is quite successful. The orange dots in Figure 6 show the evolution of the droplet jumping height after 3 successive impacts on the superhydrophobic substrate when an AC pulse of 200 V was precisely applied during the spreading phase. With the help of the droplet capacitance sensor, we were able to synchronize the electric actuation with the spreading of the droplet on the substrate, in the way that electrowetting was only applied in the spreading phase of the impact but not in the recoiling phase. This cleverly avoids the competition of electrowetting with inertia when the droplet base area starts to decrease in the latter stage of impact. By applying electrowetting to the droplet every time it hits and spreads on the substrate, the droplet experiences a downward pulling force which makes it spread faster on the substrate (please refer to File S5 of Supplementary Material), and therefore, the droplet gains additional momentum during each impacting event, which enables it to jump higher after detaching from the substrate. This is indeed very similar to the pressure-driven trampolining as reported by Schutzius et al. [3]. The only difference lies in that the latter employed a pushing force (from over pressure) in the recoiling phase of the impact, while this study utilizes a pulling force in the spreading phase. The droplet contact time ($t_{c}$), according to previous studies, is defined by the balance between capillarity and inertia as $t_{c}=\sqrt{m/\gamma }$. For a spherical droplet with a radius of 0.62 mm, $t_{c}\sim$3.8 ms. This is approximately the same as calculated from the Rayleigh method ($t_{c}\sim$4 ms). As pointed by Okumura et al. [49]., the experimentally [18] observed value of $t_{c}$ should vary with the impacting speed and decrease as the trampolining process continues. In our experiment, the variation of $t_{c}$ with the impacting speed is within 20%.

### 3.3. Modeling of EW-Driven Droplet Trampoline

In order to describe the droplet trampolining behavior, we divide the droplet motion into the flight phase and the impacting phase. The droplet only experiences the gravitational force during flight phase (rising and falling in air), while it experiences a force that is periodically applied during the spreading phase of the impact. Therefore, we adopt the model developed by Schutzius et al. [3] and describe the trampolining droplet as a standard mass-spring-damper system with a forcing function. During the flight phase, the position of the droplet centroid is governed by projectile motion, which is described by

(8) $$y=y_{0}+v_{0}t-\frac{1}{2}gt^{2}\;\mathrm{if}\;y\geq R_{0}$$

where $y_{0},$ $v_{0}$ and t represent the initial centroid position, initial velocity and time. During the spreading phase of the impact, the droplet experiences a downward pulling force $F^{\prime }$ from the electrowetting effects, which we write as

(9) $$\frac{F^{\prime }}{mg}\sim \frac{\Delta R}{y_{0}-R_{0}}\sim \left(1-\frac{1}{{\left(1+\eta \right)}^{2}}\right)$$

where $R_{0}$ and ΔR represent the equivalent radius of the droplet and the deformation caused by $F^{\prime }$, and η is the electrowetting number calculated from Equation (1).

Equation (9) is extracted from the cylindrical model we developed before [8]. By referring to Schutzius et al. [3] and modifying it, the motion of the droplet can be described as

(10) $$m\frac{d^{2}y}{dt^{2}}+c\frac{dy}{dt}+f_{k}\left(y\right)=F\left(t\right)\;\mathrm{if}\;y<R_{0}$$

where $f_{k}$ is the force due to stiffness of the droplet, and *c* is the damping ratio. The solutions of Equations (8) and (10) are equivalent to

(11) $$\left\{\begin{array}{c} v\left(t\right)=v_{0}+\int_{0}^{t}a\left(\tau \right)d\tau \;\\ y\left(t\right)=y_{0}+\int_{0}^{t}v\left(\tau \right)d\tau \;\\ a\left(t\right)=-g,\;y\geq R_{0}\;\\ a\left(t\right)=-\frac{F}{m},\;F=-k\delta -cv-mg,\;\frac{dy}{dt}>0,\;y\left(t\right)<R_{0}\\ a\left(t\right)=-\frac{F}{m},\;F=-k\delta -cv-mg\left(2-\frac{1}{{\left(1+\eta \right)}^{2}}\right),\;\frac{dy}{dt}<0,\;y\left(t\right)<R_{0}\; \end{array}\right.$$

where $a\left(t\right),\;v\left(t\right)$ and $y\left(t\right)$ represent the acceleration, velocity and position of the droplet, and τ is time. k represents the stiffness of the droplet, which is roughly proportional to the surface tension γ of the droplet, and δ represents the deformation of the droplet. The third to fifth row of Equation (11) mark the flying, spreading and receding phases of the droplet. Since the capacitor formed between the droplet and the substrate is directly related to the velocity of the droplet (e.g.,$\;\frac{dy}{dt}=-g$ indicates that the value of the capacitor is constant and close to zero; $\frac{dy}{dt}<0,\;y\left(t\right)<R_{0}$ marks the increasing of the capacitor value and therefore the spreading phase, and $\frac{dy}{dt}>0,\;y\left(t\right)<R_{0}$ marks the decreasing of the capacitor value and therefore the recoilind phase), the difference between different conditions can also be expressed as a function of the capacitance. According to the cylindrical model we developed before, ky can be written as

(12) $$ky=\gamma \;\left[{\left(1-0.5\eta \right)}^{2/3}-1\right]R_{0}$$

We take $\mathrm{Oh}=\mu {\left(\gamma \rho R_{0}\right)}^{-1/2}$ as the damping ratio [50] and $\tau^{\prime }=\sqrt{m/\gamma }$ as the inertial-capillary timescale. Since the system is clearly underdamped, the damping ratio is ~0.09. The initial deformation of the droplet resting on the superhydrophobic substrate can be calculated from

(13) $$\left\{\begin{array}{c} \;\delta_{0}=\frac{y_{0}-R_{0}}{R_{0}}\\ \gamma \delta_{0}R_{0}\sim mg \end{array}\right.$$

where $R_{0}$ is the radius of the equivalent sphere, $\delta_{0}$ is the deformation ratio of the droplet relative to $R_{0}$. The initial position of the center of mass of the droplet is $y_{0}=\left(1+\delta_{0}\right)R_{0}$, and the initial velocity of the droplet is obviously $v_{0}=0$.

With the values of $y_{0}$, $v_{0}$ and the expression for $F^{\prime }\left(t\right)$, the solution to Equations (8) and (10) (according to the third to fifth row of Equation (11)) is plotted in Figure 6 together with the experimental data. Except for a discrepancy between the jumping height, a good agreement is found between the two. As to the effect of voltage level on the trampolining height, it is natural that they should follow a similar trend as reflected by File S4 of the Supplementary Material, which should also agree with the prediction of the trampoline model discussed before, since a larger voltage level represents a larger driving force *F’* according to Equations (1) and (9). Therefore, this part of the work is skipped here. For a discussion on the velocity and acceleration of the droplet, please refer to File S5 of the Supplementary Materials.

## 4. Conclusions

In summary, this study has proposed a method for trampolining a droplet on superhydrophobic surfaces using electrowetting. With the feedback information provided by real-time capacitor sensing, we were able to apply EW to the impacting droplet in a precise manner. Since EW pulse is applied periodically in the spreading phase of the droplet, the droplet gains additional momentum after each impact and displays a trampolining behavior. We have modeled the droplet trampolining process using a periodically driven damped harmonic oscillator, and the experiments show sound agreement with theoretical predictions. The active method proposed and the findings from this study will offer valuable insights for applications that demand vertical transportation of the droplets between chips arranged in parallel or detachment of droplets from solid surfaces.

## Supplementary Materials

The following supporting information can be downloaded at: https://www.mdpi.com/article/10.3390/mi13030345/s1, ESI 4: Droplet base radius as a function of time for actuation pulses of different duration; Video 3: 400V_8ms; Video 1: jumping and damping-300V-5KF per second; Figure S3: Drop base diameter (Db) evolution with time (X) from different pulse duration of 400V for (a) in air, 3, 4, 6, 8, 10, 12 and 13 ms; Video 2: trampolining-200V-2KF per second; Figure S5: Experimental values of droplet velocity (dark line) and acceleration (pink line) as a function of time for the droplet trampolining on a superhydrophobic surface, driven by electrowetting force applied during the spreading phase of each impact cycle. The acceleration has been normalized by the gravitational acceleration g; ESI 6: Droplet centroid velocity and acceleration duration trampolining process.

## References

[1] Wisdom K.M., Watson J.A., Qu X., Liu F., Watson G.S., Chen C.-H. (2013). Self-cleaning of superhydrophobic surfaces by self-propelled jumping condensate. Proc. Natl. Acad. Sci. USA.

[2] Krupenkin T., Taylor J.A. (2011). Reverse electrowetting as a new approach to high-power energy harvesting. Nat. Commun..

[3] Schutzius T.M., Jung S., Maitra T., Graeber G., Köhme M., Poulikakos D. (2015). Spontaneous droplet trampolining on rigid superhydrophobic surfaces. Nature.

[4] Boreyko J.B., Chen C.-H. (2009). Self-propelled dropwise condensate on superhydrophobic surfaces. Phys. Rev. Lett..

[5] Bormashenko E., Pogreb R., Whyman G., Bormashenko Y., Erlich M. (2007). Vibration-induced Cassie-Wenzel wetting transition on rough surfaces. Appl. Phys. Lett..

[6] Chen R., Jiao L., Zhu X., Liao Q., Ye D., Zhang B., Li W., Lei Y., Li D. (2018). Cassie-to-Wenzel transition of droplet on the superhydrophobic surface caused by light induced evaporation. Appl. Therm. Eng..

[7] Liu G., Fu L., Rode A.V., Craig V.S. (2011). Water droplet motion control on superhydrophobic surfaces: Exploiting the Wenzel-to-Cassie transition. Langmuir.

[8] Wang Z., Ende D.V.D., Pit A.M., Lagraauw R., Mugele F. (2017). Jumping drops on hydrophobic surfaces, controlling energy transfer by timed electric actuation. Soft Matter.

[9] Sourais A.G., Papathanasiou A.G. (2021). Modelling of Electrowetting-Induced Droplet Detachment and Jumping over Topographically Micro-Structured Surfaces. Micromachines.

[10] Mugele F., Baret J.-C. (2005). Electrowetting: From basics to applications. J. Phys. Condens. Matter.

[11] Moon H., Cho S.K., Garrell R.L., Kim C.-J.C. (2002). Low voltage electrowetting-on-dielectric. J. Appl. Phys..

[12] Pollack M.G., Fair R.B., Shenderov A.D. (2000). Electrowetting-based actuation of liquid droplets for microfluidic applications. Appl. Phys. Lett..

[13] Berge B., Peseux J. (2000). Variable focal lens controlled by an external voltage: An application of electrowetting. Eur. Phys. J. E.

[14] Hayes R.A., Feenstra B.J. (2003). Video-speed electronic paper based on electrowetting. Nature.

[15] Cho S.K., Moon H., Kim C.-J. (2003). Creating, transporting, cutting, and merging liquid droplets by electrowetting-based actuation for digital microfluidic circuits. J. Microelectromech. Syst..

[16] Lee S.J., Lee S., Kang K.H. (2012). Droplet jumping by electrowetting and its application to the three-dimensional digital microfluidics. Appl. Phys. Lett..

[17] Raman K.A., Jaiman R.K., Lee T.-S., Low H.-T. (2016). A numerical study on electrowetting-induced jumping and transport of droplet. Int. J. Heat Mass Transf..

[18] Zhang K., Li Z., Chen S. (2019). Analytical prediction of electrowetting-induced jumping motion for droplets on hydrophobic substrates. Phys. Fluids.

[19] Burkhart C.T., Maki K.L., Schertzer M.J. (2020). Coplanar Electrowetting-Induced Droplet Detachment from Radially Symmetric Electrodes. Langmuir.

[20] Hong J., Lee S.J. (2015). Detaching droplets in immiscible fluids from a solid substrate with the help of electrowetting. Lab A Chip.

[21] Merdasi A., Moosavi A., Shafii M. (2019). Electrowetting-induced droplet jumping over topographically structured surfaces. Mater. Res. Express.

[22] Weng N., Wang Q., Gu J., Li J., Wang C., Yao W. (2021). The dynamics of droplet detachment in reversed electrowetting (REW). Colloids Surf. A Physicochem. Eng. Asp..

[23] Xiao K., Wu C.-X. (2021). Curvature effect of electrowetting-induced droplet detachment. J. Appl. Phys..

[24] Hong J., Kim Y.K., Won D.J., Kim J., Lee S.J. (2015). Three-dimensional digital microfluidic manipulation of droplets in oil medium. Sci. Rep..

[25] Wang Q., Li L., Gu J., Zhang C., Lyu J., Yao W. (2021). Manipulation of a Nonconductive Droplet in an Aqueous Fluid with AC Electric Fields: Droplet Dewetting, Oscillation, and Detachment. Langmuir.

[26] Vo Q., Tran T. (2021). Droplet ejection by electrowetting actuation. Appl. Phys. Lett..

[27] Lee S.J., Lee S., Kang K.H. (2011). Jumping of a droplet on a superhydrophobic surface in AC electrowetting. J. Vis..

[28] Vo Q., Tran T. (2019). Critical conditions for jumping droplets. Phys. Rev. Lett..

[29] Lee S.J., Hong J., Kang K.H., Kang I.S., Lee S.J. (2014). Electrowetting-induced droplet detachment from hydrophobic surfaces. Langmuir.

[30] Cavalli A., Preston D.J., Tio E., Martin D.W., Miljkovic N., Wang E.N., Blanchette F., Bush J.W. (2016). Electrically induced drop detachment and ejection. Phys. Fluids.

[31] Islam M.A., Tong A.Y. (2018). A numerical study on electrowetting-induced droplet detachment from hydrophobic surface. J. Heat Transf..

[32] Lapierre F., Coffinier Y., Boukherroub R., Thomy V. (2013). Electro-(de)wetting on Superhydrophobic Surfaces. Langmuir.

[33] Gong J. (2008). All-electronic droplet generation on-chip with real-time feedback control for EWOD digital microfluidics. Lab A Chip.

[34] Chen J.Z., Darhuber A.A., Troian S.M., Wagner S. (2004). Capacitive sensing of droplets for microfluidic devices based on thermocapillary actuation. Lab A Chip.

[35] Shih S.C., Fobel R., Kumar P., Wheeler A.R. (2011). A feedback control system for high-fidelity digital microfluidics. Lab A Chip.

[36] Murran M.A., Najjaran H. (2012). Capacitance-based droplet position estimator for digital microfluidic devices. Lab A Chip.

[37] Bhattacharjee B., Najjaran H. (2012). Droplet sensing by measuring the capacitance between coplanar electrodes in a digital microfluidic system. Lab A Chip.

[38] Pham J.T., Paven M., Wooh S., Kajiya T., Butt H.-J., Vollmer D. (2017). Spontaneous jumping, bouncing and trampolining of hydrogel drops on a heated plate. Nat. Commun..

[39] Graeber G., Regulagadda K., Hodel P., Küttel C., Landolf D., Schutzius T.M., Poulikakos D. (2021). Leidenfrost droplet trampolining. Nat. Commun..

[40] Liu M., Du H., Cheng Y., Zheng H., Jin Y., To S., Wang S., Wang Z. (2021). Explosive Pancake Bouncing on Hot Superhydrophilic Surfaces. ACS Appl. Mater. Interfaces.

[41] Gilet T., Bush J.W. (2009). The fluid trampoline: Droplets bouncing on a soap film. J. Fluid Mech..

[42] Ryu J., Kim K., Park J., Hwang B.G., Ko Y., Kim H., Han J., Seo E., Park Y., Lee S.J. (2017). Nearly perfect durable superhydrophobic surfaces fabricated by a simple one-step plasma treatment. Sci. Rep..

[43] Yi U.C., Kim C.J. (2006). Characterization of electrowetting actuation on addressable single-side coplanar electrodes. J. Micromech. Microeng..

[44] Bostwick J., Steen P. (2009). Capillary oscillations of a constrained liquid drop. Phys. Fluids.

[45] Vejrazka J., Vobecka L., Tihon J. (2013). Linear oscillations of a supported bubble or drop. Phys. Fluids.

[46] Miller C., Scriven L. (1968). The oscillations of a fluid droplet immersed in another fluid. J. Fluid Mech..

[47] Boreyko J.B., Chen C.H. (2010). Self-propelled jumping drops on superhydrophobic surfaces. Phys Fluids.

[48] Enright R., Miljkovic N., Sprittles J., Nolan K., Mitchell R., Wang E.N. (2014). How Coalescing Droplets Jump. Acs Nano.

[49] Okumura K., Chevy F., Richard D., Quéré D., Clanet C. (2003). Water spring: A model for bouncing drops. EPL (Europhys. Lett.).

[50] Hong J., Kim Y.K., Kang K.H., Oh J.M., Kang I.S. (2013). Effects of drop size and viscosity on spreading dynamics in DC electrowetting. Langmuir.

